# Attentional and physiological processing of food images in functional dyspepsia patients: A pilot study

**DOI:** 10.1038/s41598-017-19112-0

**Published:** 2018-01-23

**Authors:** In-Seon Lee, Hubert Preissl, Katrin Giel, Kathrin Schag, Paul Enck

**Affiliations:** 1 Department of Internal Medicine VI: Psychosomatic Medicine and Psychotherapy, University of Tübingen, Tübingen, Germany; 2 IMPRS for Cognitive and Systems Neuroscience, Tübingen, Germany; 3 Institute for Diabetes Research and Metabolic Diseases of the Helmholtz Center Munich at the University of Tübingen, Germany Center for Diabetes Research (DZD), Department of Internal Medicine IV, Tübingen, Germany; 4 Department of Pharmacy and Biochemistry, Institute of Pharmaceutical Sciences, University of Tübingen, Tübingen, Germany; 5 Institute for Diabetes and Obesity, Helmholtz Diabetes Center at Helmholtz Zentrum München, German Research Center for Environmental Health (GmbH), Neuherberg, Germany

## Abstract

The food-related behavior of functional dyspepsia has been attracting more interest of late. This pilot study aims to provide evidence of the physiological, emotional, and attentional aspects of food processing in functional dyspepsia patients. The study was performed in 15 functional dyspepsia patients and 17 healthy controls after a standard breakfast. We measured autonomic nervous system activity using skin conductance response and heart rate variability, emotional response using facial electromyography, and visual attention using eyetracking during the visual stimuli of food/non-food images. In comparison to healthy controls, functional dyspepsia patients showed a greater craving for food, a decreased intake of food, more dyspeptic symptoms, lower pleasantness rating of food images (particularly of high fat), decreased low frequency/high frequency ratio of heart rate variability, and suppressed total processing time of food images. There were no significant differences of skin conductance response and facial electromyography data between groups. The results suggest that high level cognitive functions rather than autonomic and emotional mechanisms are more liable to function differently in functional dyspepsia patients. Abnormal dietary behavior, reduced subjective rating of pleasantness and visual attention to food should be considered as important pathophysiological characteristics in functional dyspepsia.

## Introduction

Functional dyspepsia (FD) is defined as a disorder with unexplained symptoms such as postprandial fullness, early satiation, epigastric pain and burning that originate in the gastroduodenal region^1,2^. So far, our knowledge of pathophysiological abnormalities in FD has been limited to functional abnormalities in the gastrointestinal tract (visceral hypersensitivity, abnormal accommodation, delayed gastric emptying, and gastric dysmotility), and only a small number of studies have investigated the psychological characteristics of FD patients and revealed the crucial role of anxiety, depression, and somatization^3–5^.

More recent studies investigating the role of dietary habit and nutritional intake in FD patients suggest that fat ingestion is a factor in symptom triggering^6–8^. Although it is already known that FD patients tolerate only small amounts of food, evidence on the extent of nutritional intake of daily meals remains inconclusive^9^. One of the limitations of previous studies in FD patients was that a food diary or questionnaire was used to measure their dietary habits, possibly causing a recall bias^10–12^. In studies using dietary interventions in FD patients, specific amounts of solid or liquid type meals were served to determine the meal-related dyspeptic symptom, gastric accommodation, or hormonal changes^13–16^. Furthermore, the psychophysiological response and cognitive processing of food stimuli in FD patients are not well established despite the fact that these are important determinants in the pathophysiology of eating disorders such as anorexia nervosa, binge eating disorder, and obesity^17,18^. Since a close relationship between the exacerbated FD symptoms and meal ingestion has been reported^13–16^, chronic negative experience of eating may cause abnormal behavioral and cognitive response, i.e., avoidance or an aversive response rather than a positive approach to food stimuli.

Visual food stimuli have been used to investigate food-related behavior in patients with obesity^19^, anorexia nervosa^20^, and binge eating disorder^21^. Food images are generally used as pleasure or incentive stimuli, eliciting a positive response or causing an attentional bias^22^. Since previous episodes of the stimuli (food) and conditioning of the stimuli with somatic symptoms may affect the attention to stimuli^22,23^, visual attention to food in FD patients might be reduced in comparison to healthy controls (HC).

Eye tracking, which measures the gaze parameters such as initial fixation and total duration of fixation on images^24^, is well suited to the investigation of initial saliency and the later cognitive processing of images. In addition, autonomic nervous system function and facial muscle activity can provide further support for altered homeostatic and emotional changes during food image processing in FD patients. Skin conductance response (SCR) and heart rate variability (HRV) have been used as the parameter of the arousal level of the sympathetic branch of autonomic nervous system and of the balance of sympathetic and parasympathetic activity, respectively. Electromyography (EMG) of facial muscles measuring the intensity of the contraction of the corrugator supercilii and zygomaticus major muscle has been used to quantify negative and positive facial emotional response, respectively^25–27^.

In the current study, we aimed to determine the physiological/emotional response and visual attention of FD patients to food images. We evaluated the physiological, emotional, and attentional response of FD patients to high fat food, low fat food, and non-food images after taking an ad-libitum breakfast. We hypothesized that, in comparison to HC, 1) FD patients consume a smaller amount of food, but have higher dyspeptic symptoms afterwards; 2) FD patients show negative valence and increased arousal level to food images, particularly to high fat food images; 3) FD patients show decreased visual attention to food images, especially to high fat food images.

## Results

### Sample characteristics

Sample characteristics and scores of questionnaires are presented in Table 1. Beck Depression Inventory (BDI), Food Craving Questionnaire (FCQ)-state score, age of HC group, and body mass index (BMI) of FD group were not normally distributed (*P* < 0.05). No significant differences in age and BMI between groups (*P* > 0.5) was shown. Eleven FD patients showed both postprandial distress syndrome (PDS) and epigastric pain syndrome (EPS) (9 females), 3 patients (all females) had PDS only, and 1 patient (male) had EPS only. FD patients showed a significantly higher Nepean Dyspepsia Index (NDI)_Symptom score (*P* < 0.001) and a lower NDI_QOL score (*P* < 0.001) than HC group. FD patients also showed significantly higher depression and anxiety levels (*P* < 0.01) and higher FCQ-state score (*P* < 0.05) than HC. The total and subscale scores in Eating Disorders Examination questionnaire (EDE-Q) and Fat Preference Questionnaire (FPQ) did not differ significantly between groups.

**Table 1:** Baseline characteristics of the study sample.

	Healthy controls	FD patients	P value
Gender (m/f)	5/12	3/12	—
Subgroup	—	PDS: 2/12, EPS: 3/9	—
Age (year)	37.65 ± 4.02	41 ± 4.72	NS*
BMI (kg/m^2^)	24.95 ± 0.73	23.27 ± 1.19	NS*
NDI_Symptom	10.56 ± 1.90	70.62 ± 9.51	*P* < 0.001
NDI_QOL	46.8 ± 1.35	23.62 ± 2.33	*P* < 0.001
EDE-Q Total	1.25 ± 0.26	1.05 ± 0.29	NS
Restraint scale	1.07 ± 0.28	0.84 ± 0.23	NS
Eating concern	0.33 ± 0.14	0.31 ± 0.15	NS
Weight concern	1.31 ± 0.32	1.13 ± 0.34	NS
Shape concern	1.58 ± 0.35	1.45 ± 0.33	NS
BDI-II	3.94 ± 1.61	9.77 ± 2.44	*P* < 0.01*
STAI_state	31.06 ± 1.64	43.46 ± 3.21	*P* < 0.01
STAI_trait	31.81 ± 2.06	44.54 ± 3.34	*P* < 0.01
FCQ_state	31.94 ± 3.09	42.93 ± 3.31	*P* < 0.05*
FCQ_trait	83.94 ± 7.28	92.08 ± 9.90	NS
FPQ_TASTE (%)	55.89 ± 5.26	65.98 ± 4.81	NS
FPQ_FREQ (%)	52.10 ± 5.05	57.26 ± 4.83	NS
FPQ_DIFF (%)	3.79 ± 1.73	8.72 ± 2.23	NS

Mean ± standard error; Higher score indicates more severe symptom (NDI_symptom), depression (BDI-II), anxiety (STAI), craving for food (FCQ), better quality of life (NDI_QOL), and higher frequency of eating concerns (EDE-Q).

BDI: Beck depression inventory (range: 0–63); BMI: body mass index; EDE-Q: Eating disorder examination questionnaire (range: 0–6); EPS: epigastric pain syndrome; f: female; FCQ: Food cravings questionnaire (range: 15–75 for state, 39–234 for trait); FD: functional dyspepsia; FPQ: Fat preference questionnaire; FPQ_TASTE: how much better high fat food tastes, FPQ_FREQ: how much high fat food eaten more often, FPQ_DIFF: high fat restriction (FPQ_TASTE-FPQ_FREQ); m: male; NDI: Nepean dyspepsia index (range: 0–195); NS: statistically not significant; PDS: postprandial distress syndrome; QOL: quality of life (range: 0–124); STAI: State trait anxiety inventory (range: 0–80).

P value: two sample t-test FD vs HC.

*P value: Mann Whitney-U test FD vs HC.

### Food/energy intake and FD symptoms

Following overnight fasting, FD patients ate significantly less bread than HC group (FD: 61.6 ± 5.07 g; HC: 76.71 ± 6.95 g, *P* < 0.05). Albeit FD patients consumed less fat (FD: 9.25 ± 1.12 g; HC: 10.38 ± 1.63 g), carbohydrate (FD: 51.85 ± 6.61 g; HC: 62.53 ± 6.60 g), and protein (FD: 8.56 ± 1.64 g; HC 9.27 ± 1.36 g) than HC group, these differences were statistically not significant. On the whole, patients in the FD group consumed significantly less total energy than HC group (FD: 332.19 ± 37.77 kcal; HC 389.76 ± 38.03 kcal, *P* < 0.05).

FD symptom ratings of baseline, and at three time points after ingestion (Post1, Post2, Post3) are described in Supplementary Table 1. Hunger rating decreased immediately after breakfast, and increased subsequently in both groups. Appetite also decreased initially and increased gradually afterwards, but FD patients had a significantly lower appetite at Post3 than HC (*P* < 0.05). Abdominal fullness in the FD group was only slightly higher than HC before breakfast, but significantly higher immediately after breakfast (*P* < 0.05). Significant main effects of group (FD > HC) were found for abdominal pain (*P* < 0.05), discomfort, burning, and bloating symptoms (*P* < 0.01).

### Experiment 1. Measurement of physiological response

Physiological response and pleasantness ratings of food and non-food images in FD patients and HC are summarized in Supplementary Table 2.

#### Pleasantness rating

Analysis of variance (ANOVA) for the 5 image sets showed a significant main effect of image (*P* < 0.001). In accordance with the post-hoc analysis (Bonferroni corrected), pleasantness of negative images was significantly lower than of any other images (*P* < 0.001). Pleasantness of high fat food images was significantly lower than of positive images (*P* < 0.001). In both groups, low fat food images and positive images were rated significantly higher than neutral images (*P* < 0.001). Subsequent analysis on high fat and low fat food images showed significant main effects of group and image (*P* < 0.05). Pleasantness ratings of food images in FD were significantly lower than in HC, and pleasantness of high fat food images was rated significantly lower than that of low fat food images in FD (*P* < 0.05).

#### SCR

ANOVA analysis for 5 image sets resulted in a significant main effect of image (*P* < 0.001). Post-hoc analysis (Bonferroni corrected) showed that, in both groups, SCR standardized ratio for negative images was significantly higher than for other images (vs neutral, positive, high fat images, each *P* < 0.001; vs low fat images *P* < 0.01). There were no significant differences between groups for either ANOVA.

#### EMG corrugator supercilii

ANOVA analysis for 5 image sets showed a significant main effect of image (*P* < 0.001). Post-hoc analysis (Bonferroni corrected) showed that the EMG response to negative images was significantly higher than to any other image (positive, high fat food, low fat food images, all *P* < 0.001; neutral image *P* < 0.01). There were no significant differences between groups from either ANOVA.

#### EMG zygomaticus major

ANOVA analysis for 5 image sets showed that there was a significant main effect of image (*P* < 0.001) and interaction of group*image (*P* < 0.05). Post-hoc analysis (Bonferroni corrected) showed that the zygomaticus major muscle EMG response was significantly higher to high fat food images than to negative (*P* < 0.01) and low fat food images (*P* < 0.05). EMG signal was significantly higher to positive images than to negative, neutral, and low fat food images (all *P* < 0.001). No differences were found between groups from the 2 × 5 ANOVA. A 2 × 2 ANOVA analysis for high fat and low fat images showed a significant main effect of image (*P* < 0.01). EMG activation was lower in FD patients than in HC and significantly higher for high fat food images than for low fat food images in HC (*P* < 0.01).

#### HRV SDNN

2 × 5 ANOVA analysis showed that there was a main effect of group (p < 0.05), and FD patients showed higher SDNN (Standard Deviation of Normal-to-Normal interbeat intervals reflecting the overall variation) values than HC group.

#### HRV HF

No significant main effect was registered for either the group for the images of amplitude of high frequency band (HF) value.

#### HRV LF/HF ratio

2 × 5 ANOVA analysis showed that there was a significant main effect of group (*P* < 0.05), and FD patients showed significantly lower LF/HF ratio (the ratio of amplitude of low frequency band (LF) and amplitude of high frequency band (HF) values) than HC group.

### Experiment 2. Eye tracking experiment

#### Initial fixation (coefficient %)

**Figure 1:**
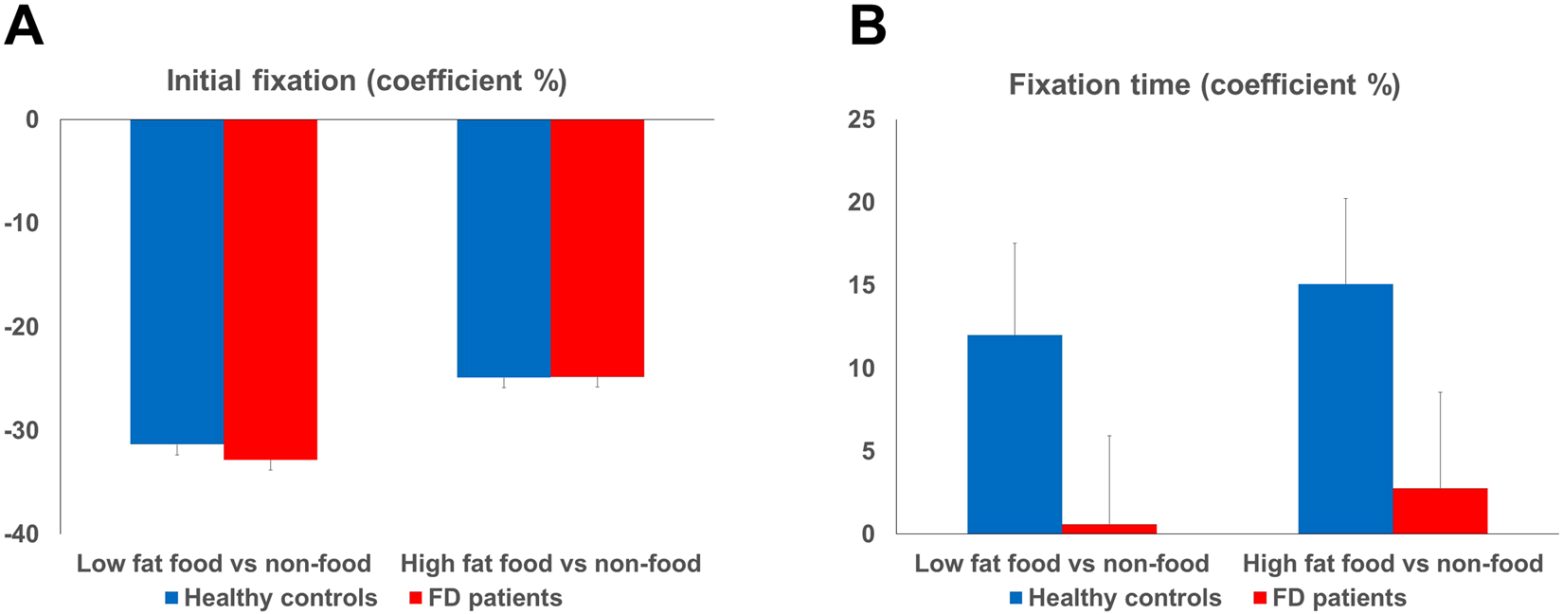
Coefficient percentage (%) of initial fixation and total fixation time (ms) in FD and healthy controls. Mean and standard error of coefficient % of initial fixation (**A**) and total fixation duration (**B**) on low fat food and high fat food images compared to paired non-food images in FD patients and healthy controls. There were no significant differences of initial fixation between groups and images. Total fixation time was significantly lower in FD patients than in HC for both high and low fat food images (*P* < 0.05).

There were no significant differences according to the ANOVA (high fat: FD −24.78 ± 7.53, HC −24.87 ± 6.19; low fat: FD −32.80 ± 5.62, HC −31.33 ± 3.86, Fig. 1A)

#### Fixation duration (coefficient %)

There was a significant main effect of group and both high and low fat food images were fixated significantly less often by FD patients than by HC (high fat: FD 2.77 ± 5.18, HC 15.07 ± 5.16; low fat: FD 0.60 ± 5.34, HC 12.01 ± 5.53; *P* < 0.05, Fig. 1B).

#### Anticipated symptom rating

There was a significant main effect of group on anticipated symptom rating, with FD patients showing higher ratings to high fat food images used in Experiment 2 than HC (*P* < 0.001). Post-hoc analysis (Bonferroni corrected) showed that FD patients anticipated significantly higher pain and burning sensation than HC group (*P* < 0.05, *P* < 0.01, respectively) and that there were no differences in fullness and satiation between groups. With regard to the low fat food images, none of the symptoms differed between groups (Supplementary Table 3).

### Correlation analysis

Correlation analysis revealed significant negative correlations between the fat intake and BDI-II (non-parametric Spearman correlation, r = −0.62), fat intake and FPQ_DIFF (r = −0.93), energy intake and FPQ_DIFF (Pearson correlation, r = −0.95, *P* < 0.05) in FD patients.

## Discussion

We investigated the physiological responses and visual attention to food and non-food images in FD patients and healthy controls. Food craving, depression, and anxiety scores were significantly higher in FD patients than in HC. Despite lower total food/energy (kcal) consumption than HC group, FD patients reported more symptoms of bloating, nausea, vomiting, abdominal pain, abdominal discomfort, and burning sensation after food intake. Furthermore, FD patients showed significantly less pleasantness ratings for both high and low fat food images than HC group. Although there was no difference in the initial orientation bias between groups, FD patients had a significantly lower total attentional processing time of food images versus non-food images than HC group. The depression score with the consumption of fat, and fat restriction score with fat/total energy intake were negatively correlated in FD patients only.

In this study, FD patients showed higher meal-induced FD symptoms after consuming less food and energy than healthy controls. It is noteworthy that the pain and burning sensation in FD patients decreased immediately after meal ingestion and then gradually increased again. These results suggest that food ingestion can not only aggravate but also temporarily alleviate FD symptoms. According to a previous study^14^, the intensity of each FD symptom increased significantly following meal ingestion. These inconsistent results may be due to the different composition of meals and instructions (“eat everything” vs “eat as much as you want”), and sample characteristics. Furthermore, we observed that FD patients also suffered from FD symptoms (pain, discomfort, burning, bloating) even when they were in a fasted state. FD patients are known to eat more frequently, but they take smaller portions and are unable to finish a normal meal portion. This may be due to dynamic changes of symptoms in a state of hunger or fullness.

As often reported in earlier studies^4,28–30^, FD patients had significantly higher anxiety and depression levels than HC group. In the current study we detected a negative correlation between the depression score and the amount of fat intake in FD patients. Chronic food restriction induced depression-like behavior in rats^31^ and high fat diet showed an anti-depressive effect in mice^32^. Fat intake was negatively correlated to depression score^33^ and a cohort study in the population without depression found that trans unsaturated fatty acids intake was positively related to the incidence of depression^34^. Although a clear conclusion cannot be drawn from correlation analyses, the results may show the mutual influences of a psychological state and eating behavior in FD patients.

High HRV and decreased sympathetic activation in FD patients were observed regardless of picture category. This could therefore be an intrinsic characteristic of FD patients rather than a response to external stimuli. Furthermore, the emotional response during the visual stimulation of food and non-food cues did not differ significantly between groups. This can be interpreted along with the eye tracking results. FD patients showed a tendency of initial attention (a marker of an immediate attention-grabbing effect of images) similar to that of HC group and a lower total attention processing time (fixation duration, a marker of a total attention-grabbing effect) for food images. While visual food images may not immediately induce negative valence and avoidance responses, a late cognitive processing of the images by higher cognitive function may cause the avoidance response to food images in FD patients while processing food images. While still hypothetical, it is nonetheless plausible that high level cognitive functions rather than autonomic and emotional mechanisms can operate differently in FD patients. Furthermore, a decreased fixation duration on food images in FD patients is not in line with earlier findings in patients with obesity and binge eating disorder^35,36^ (where increased duration on food images was reported) and is similar to the results from anorexia nervosa patients^37^. This might indicate a positive or negative perception of food cues in eating-related diseases.

The reduced pleasantness of and attentional bias to visual food stimuli in FD patients could be considered in future psychotherapeutic intervention and research. Various treatment options have been proposed for FD, such as H. pylori eradication, prokinetic agents, acid suppressive medications, and antidepressants. Nevertheless, a standardized treatment strategy for FD patients has yet to be established and cognitive behavioral therapy remains an unexplored area^38,39^. A new therapy that includes self-restraint response to food, emotional management, and eating behavior modification could be considered for patients who do not respond to conventional therapies. Furthermore, how FD patients perceive, encode, store, and recall the value of food and how food memories influence their food-related decision making might be interesting topics for future studies in FD mechanisms.

In the interviews conducted before the study, almost all FD patients complained about the changes in their eating behavior and their poor quality of life. Most patients avoided symptom-related foods, such as fatty foods, bread, pasta, or alcohol. The list of foods avoided varied from person to person. Fatty foods aggravated the symptoms in some patients, whereas others remained unaffected. Nevertheless, the high fat restriction score was significantly related to the lower intake of fat and total energy in FD patients only, and they anticipated more severe symptoms from high fat food than HC. Previous negative memory of the aftereffect of eating may affect the anticipation of symptoms by learning of stimuli and somatic symptoms. This could be extended to the restriction of food intake, reduced pleasantness rating and the attentional avoidance of food images^22,23^.

Limitations of this study were the use of food images instead of real food and subjective ratings of symptoms and pleasantness. Unlike real food, food images lack odor and texture information. Furthermore, it was challenging to identify one particular symptom-related food item in each patient. Perceptual characteristics such as color, contrast, or brightness of image categories may differ and may influence the participants’ responses. This may be the basis for the similar autonomic and emotional responses to high fat and low fat images in our study. Secondly, our sample size was not large enough to conduct further subgroup analysis and we did not examine any differences between PDS and EPS, patients with severe and mild FD/depression/anxiety symptoms. The small sample size in this study limits the interpretation of our findings. Nevertheless, the investigation of eating behavior using standardized meals and of physiological responses using visual food cues provides new insight into pathomechanism in FD patients. Further studies with a larger sample size are necessary to expand our knowledge of the food-related behavior, cognitive, emotional, and physiological responses in FD patients.

In conclusion, we observed an increased food craving, decreased food intake, food ingestion-induced aggravation of FD symptoms, reduced food image processing time, and reduced perception of food-related pleasantness in FD patients. The effectiveness of conventional therapies in FD patients might be enhanced by the modification of psychological responses to food.

## Methods

### Participants

15 FD patients (3 male, aged 41 ± 4.72 years) and 17 age- and BMI-matched HC (5 male, aged 39.65 ± 4.02 years) were included in the study. They were all right-handed and the age range was 18–75 years and BMI (weight/height^2^) range 19–29 kg/m^2^. FD patients were diagnosed on the basis of ROME III criteria^40^ and an unsuspicious endoscopy documented in their medical records. Participants with visual impairment, severe psychiatric illness, intake of antidepressants or antipsychotics, and any food allergy or intolerance were excluded. The study was approved by the ethics committee of the Medical Faculty, Tübingen University, Germany (041/2016BO2). All participants provided informed consent and all experiments were conducted ethically according to the principles of the Declaration of Helsinki.

### Procedure

**Figure 2:**
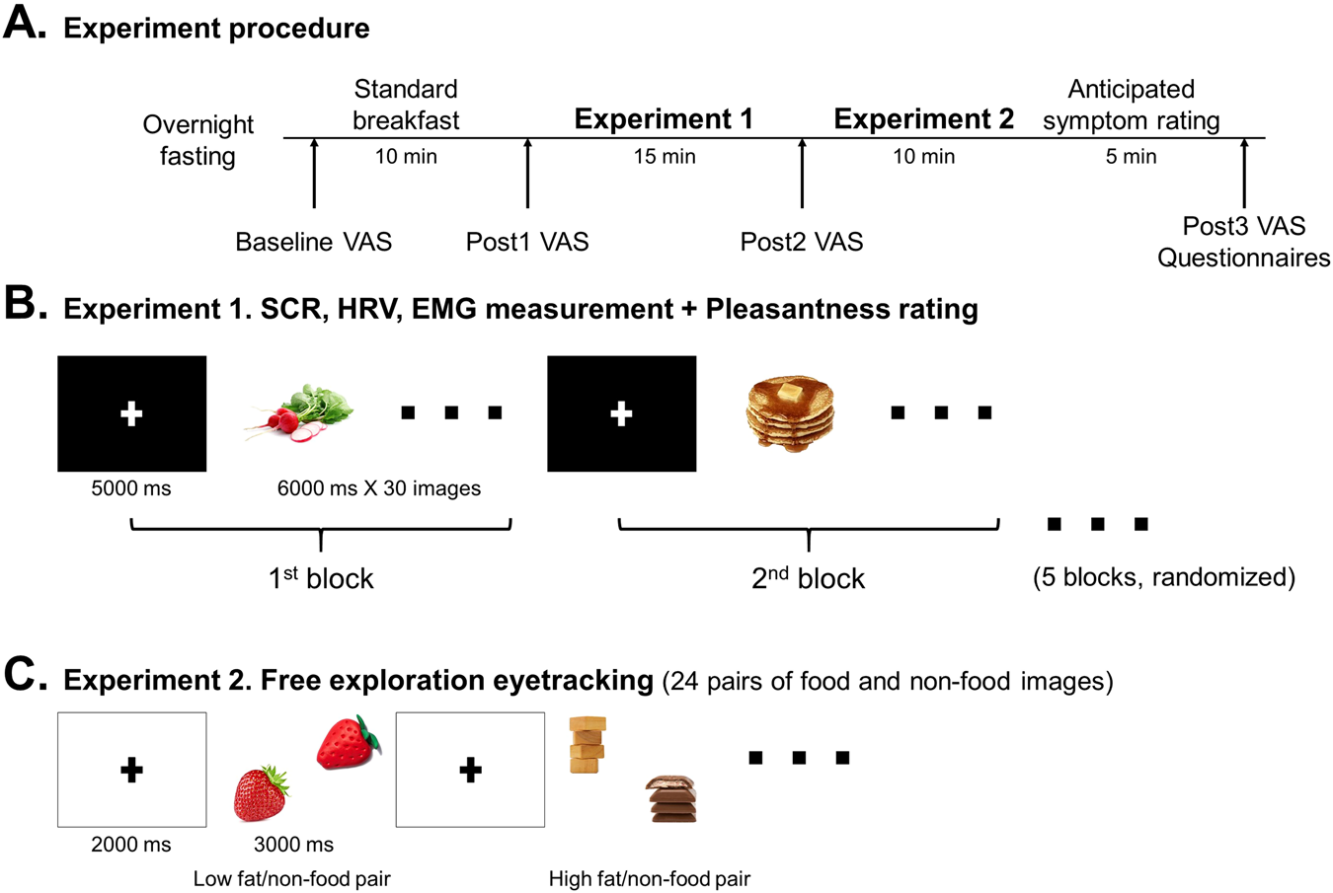
Experimental protocol. (**A**) Experimental procedure of the study. (**B**) Illustration of Experiment 1 including skin conductance response, heart rate, electromyography measurements, and pleasantness rating to food and non-food images. Randomized order of 5 blocks of images (neutral, positive, negative, high fat, and low fat food images, n = 30, 6000 ms for each image) with fixation cross (5000ms) between each block were presented. (**C**) Schematic presentation of the eye tracking experiment using free exploration paradigm. Low fat food and non-food pairs and high fat food and non-food pairs (n = 12, respectively) were presented for 3000 ms with 2000 ms of fixation cross between pairs. Location of the images (1^st^–4^th^ quadrant) was balanced.

The study was conducted at the University Clinic, Tübingen, Germany. Participants were asked to fast from 10 pm of the evening prior to the study. The study commenced at 8 a.m. the following morning. The participants began by rating their physical condition such as hunger, appetite, abdominal fullness, satiation, nausea, vomiting, abdominal pain, abdominal discomfort, burning, and bloating symptoms (baseline) on a visual analogue scale (VAS; 0 = not at all, 10 = very much). They were then served a standard breakfast consisting of bread (2 slices, 110 g), butter (36 g), jam (46 g), milk (1.5% fat, 500 ml), orange juice (500 ml), and water (total calorie 402.09 kcal, fat 14.52 g, carbohydrate 53.61 g, protein 12.98 g). The participants could eat as much as they wished within 10 minutes. VAS ratings were assessed again immediately after breakfast (Post1), between Experiment 1 and Experiment 2 (Post2, 20–25 minutes after the meal), and at the end of the experiment (Post3, 45–50 minutes after the meal, Fig. 2A). The remaining food from each participant was weighed and calorie intake was calculated.

### Experiment 1. Emotional and physiological response to food and non-food images

Skin conductance response was measured with two electrodes attached to the index and middle finger of the left hand. Three electrodes were placed on the chest region to measure the electrocardiography (ECG) signal. For facial EMG measurement, three electrodes were attached on both the corrugator supercilii and zygomaticus major muscles on the left side of the face^41^. The data were recorded with a Biopac MP36 system and Acknowledge software ver. 4.1 (Biopac Systems Inc., Goleta, USA).

Five fixed-order sets of image stimuli (neutral household items, positive, negative, high fat, and low fat food images, n = 30, respectively), were presented in a randomized order for 180 seconds (6 seconds for each image) followed by a 5-second rest with visual cross fixation between each set. Subjective pleasantness to each image was measured by pressing a button from 1 to 10 on a keyboard (1 = very unpleasant, 10 = very pleasant). Participants were also requested not to move or talk while the measurements were being carried out (Fig. 2B).

### Experiment 2. Visual attention to food and non-food images

After Experiment 1, gaze data were recorded with the eye tracking system iView X Hi-Speed (SensoMotoric Instruments GmbH, Berlin, Germany). Each participant received a standardized 13-point calibration procedure to ensure optimal gaze data quality. Following calibration, 24 pairs of images (different from those used in Experiment 1) composed of food (high fat n = 12, low fat n = 12) and non-food images (household items, n = 24), were randomly presented. Each pair of stimuli images was presented for 3 seconds and a fixation cross at the center of the screen was shown for 2 seconds between each pair. Participants were requested to freely explore the presented pictures and to fixate the cross when shown (Fig. 2C).

After the eye tracking experiment, the anticipated FD symptoms (postprandial fullness, early satiation, abdominal pain, and burning sensation) for each food image (high fat n = 12, low fat n = 12) were assessed using VAS (0 = not at all, 10 = very much) during the visual stimuli of food images without response time restriction. At the conclusion of the study, each participant’s dyspepsia symptom intensity and disease-related quality of life were assessed using the NDI^42^. Depression and anxiety levels were evaluated using the BDI-II^43^ and State-Trait Anxiety Inventory (STAI)^44^, respectively. Furthermore, the EDE-Q^45^, FCQ^46^, and FPQ^47^ were used to identify eating behavior.

### Materials and apparatus

#### Experiment 1

Positive and negative images were selected from the International affective picture system (IAPS)^48^. Taking the diversity of food and color-matching between food and non-food images into consideration, we selected neutral household items and food images from food image databases^49^. Food items for the high fat food images included cake, pancake, croissant, hamburger, cheese, pie, ham, sausage, white chocolate, peanuts, and meat. Food items for the low fat food included salad, asparagus, pea, cucumber, fruit, and rice. Images were presented and subjective pleasantness rating was recorded with Presentation® (version 16.5, www.neurobs.com). Physiological signals were recorded with a Biopac MP36 system and Acknowledge software 4.1 (Biopac Systems Inc., Goleta, USA). SCR, ECG and EMG signals were sampled at 1 kHz. For SCR, a low pass filter of 10 Hz, for ECG a bandpass filter between 0.5 and 35 Hz, and for EMG a bandpass filter between 30 and 250 Hz were applied.

#### Experiment 2

A validated image set for the eye tracking experiment was used in this study^36^. The food and non-food stimuli were subjectively matched in color and quantitatively for brightness and contrast (paired t-test, for both *P* > 0.8). The complexity, valence, and arousal levels of the images were rated in an earlier study^50^. Eye movements were recorded with the IViewX Hi-Speed and IViewX 2.8 software (SensoMotoric Instruments, Berlin, Germany) and sampling rate was set at 500 Hz.

### Data processing

#### Experiment 1

We analyzed the raw SCR signal using Ledalab software (www.ledalab.de). Raw SCR was smoothed and event-related activation was extracted when the signal exceeded 0.01muS. For the computation of the standardized ratio, the total amplitude of SCR of each block (neutral, positive, negative, high, and low fat cues) was divided by the total amplitude to normalize individual differences.

Rectified EMG was derived from raw EMG data, while integrated EMG was defined as the area under the curve of the rectified EMG signal. Muscle activation was located every 30ms automatically and visually ascertained. To calculate the standardized ratio, we divided the whole EMG signal from all muscle activations located in each block by the total amplitude (Acknowledge software 4.1, Biopac Systems Inc., Goleta, USA).

ECG signals during the visual stimuli of 5 sets of images (neutral, positive, negative, high fat, and low fat food images) were analyzed with Kubios HRV software (version 2.2, http://kubios.uku.fi/)^51^. QRS complexes were detected based on the Pan-Tompkins algorithm. The algorithm uses slope, amplitude, and width information of the QRS and comprises of two learning phases (detection threshold and RR-interval limit) and a detection phase (recognition of QRS)^52^. Missing/additional or ectopic/arrhythmic beats were visually checked and RR intervals (time interval between successive R-waves) which differed from the local average more than 0.25 seconds were identified as artifacts. Artifacts were replaced with interpolated values. SDNN (Standard Deviation of Normal-to-Normal interbeat intervals reflecting the overall variation) was computed from the artifacts-corrected series of consecutive RR intervals of each image block (6000ms). For frequency domain analysis, a linear observation model was used to detrend low frequency aperiodic trend components. Trend components were defined as ‘Z_trend_ = observation matrix*regression parameters + observation error’ and removed using the smoothness priors method (high-pass filter with a cut-off frequency: Lambda 500, f = 0.035 Hz). Frequency bands were set at 0.04–0.15 Hz for low frequency and at 0.15–0.4 Hz for high frequency. Non-parametric Fast Fourier Transform (FFT)-based power spectrum estimation was applied to calculate power spectral density of each frequency band following the cubic spline interpolation of the detrended RR series. Amplitude of low frequency band (LF), amplitude of high frequency band (HF), and the ratio of LF and HF values during the visual stimuli of images (6000ms) were calculated by using FFT algorithm and log transformed.

#### Experiment 2

The raw gaze data was analyzed using BeGaze 3.0 software (SensoMotoric Instruments GmbH, Berlin, Germany). The areas of interest (AOIs) were defined for food and non-food images and fixation cross. The initial fixation position was defined by the geographical position of gaze on AOIs and fixation duration was calculated as the sum of the duration time of fixation inside each AOI^53^. Any trials in which participants did not fixate on the cross at the onset of the trial were ruled out. Two variables were defined to test the hypothesis: 1) the coefficient percentage (%) of initial fixation on food versus non-food images: (number of initial fixations on food images–number of initial fixations on non-food images)/(number of initial fixations on food images + number of initial fixations on non-food images)*100(%), 2) the coefficient percentage (%) of total fixation duration on food versus non-food images: (fixation duration on food images–fixation duration on non-food images)/(fixation duration on food images + fixation duration on non-food images)*100(%). All authors had access to the study data and reviewed and approved the final manuscript.

### Statistical analysis

All statistical analyses were performed with IBM SPSS statistics 24.0 (IBM Corp. New York, USA). Normal distribution of data was assessed by Lilliefors-corrected Kolmogorov-Smirnov test. For normally distributed data, independent two sample t-tests were used to assess any differences between study groups (FD and HC). For data showing non-normal distribution, we used the non-parametric Mann Whitney-U test. A two-way repeated measures ANOVA with the groups (FD and HC) and the time factors (baseline, Post1, Post2, Post3) was used to identify the changes in meal-induced FD symptom ratings. A two-way ANOVA with the group (FD and HC) and type of image (2 × 5: neutral, positive, negative, high fat food, low fat food; 2 × 2: high fat, low fat food) factors were applied to the pleasantness rating, SCR, EMG, HRV variables, eye tracking data, and anticipated FD symptom rating. Two-tailed partial Pearson correlation analysis or non-parametric Spearman partial correlation were also computed between BMI, fat and total energy intake, NDI_Symptom and NDI_QOL (quality of life) scores, BDI-II, STAI, FCQ, FPQ scores, and the eye-tracking variables while controlling for age and BMI. The statistical significance level was set at α = 0.05 and a Bonferroni correction was applied to account for multiple testing if necessary.

### Data availability

All authors had full access to all the data in the study and had final responsibility for the decision to submit for publication.

### Writing assistance

English correction, funded by the European Union’s Seventh Framework Programme under REA grant agreement No. 607652 (NeuroGUT).

### Ethics approval

The study was approved by the ethics committee of the Medical Faculty, Tübingen University, Germany (041/2016BO2).

### Clinical trial registration

ClinicalTrials.gov number NCT02727556, date of registration March 21, 2016.

## Electronic supplementary material

Supplementary Dataset 1
