# Supplementary material for: Attentional and physiological processing of food images in functional dyspepsia patients: A pilot study
Source: Sci Rep. 2018 Jan 23;8:1388. doi: 10.1038/s41598-017-19112-0 (PMC5780505; doi:10.1038/s41598-017-19112-0)
Supplement: Supplementary file 1 — Supplementary Dataset 1 [file 41598_2017_19112_MOESM1_ESM.doc]

Attentional and physiological processing of food images

in functional dyspepsia patients: A pilot study

**In-Seon Lee^1,2^, Hubert Preissl^3,4^, Katrin Giel^1^, Kathrin Schag^1^, Paul Enck^1^**

1. Psychosomatic Medicine and Psychotherapy Department, University of Tübingen, Tübingen, Germany
2. IMPRS for Cognitive and Systems Neuroscience, Tübingen, Germany
3. Institute for Diabetes Research and Metabolic Diseases of the Helmholtz Center Munich at the University of Tübingen; German Center for Diabetes Research (DZD); Department of Internal Medicine IV; Department of Pharmacy and Biochemistry, Institute of Pharmaceutical Sciences, , University of Tübingen, Tübingen, Germany
4. Institute for Diabetes and Obesity, Helmholtz Diabetes Center at Helmholtz Zentrum München, German Research Center for Environmental Health (GmbH), Neuherberg, Germany

Contact information:

Prof. Dr. Paul Enck

University Hospital,

Dept. of Internal Medicine VI

Osianderstr. 5, 72076 Tübingen, Germany

Email: [paul.enck@uni-tuebingen.de](mailto:paul.enck@uni-tuebingen.de)

Short title: food study in functional dyspepsia patients

Supplementary Table 1. FD symptom ratings before and after breakfast

|  |  | **Baseline** | **Post1** | **Post2** | **Post3** | **P value (ANOVA)** |
| --- | --- | --- | --- | --- | --- | --- |
| **Hunger** | HC | 5.09±0.65 | 1.14±0.40 | 1.16±0.40 | 2.62±0.67 | main effect of time p<0.01 |
|  | FD | 4.5±0.85 | 0.71±0.28 | 1.7±0.58 | 1.75±0.42 |  |
| **Appetite** | HC | 4.68±0.52 | 1.82±0.55 | 2.31±0.60 | 3.65±0.74 | main effect of time p<0.05 |
|  | FD | 4.33±0.89 | 1.5±0.48 | 2.2±0.69 | 1.89±0.53* |  |
| **Fullness** | HC | 1.16±0.42 | 2.29±0.60 | 2.38±0.53 | 1.97±0.43 | main effect of time p<0.05 |
|  | FD | 2.67±0.86 | 4.82±0.92* | 3.03±0.69 | 3.36±0.76 |  |
| **Satiation** | HC | 1.97±0.58 | 6.54±0.59 | 5.50±0.63 | 4.71±0.74 | main effect of time p<0.01 |
|  | FD | 2.07±0.45 | 5.57±0.77 | 3.73±0.72 | 5.14±0.90 |  |
| **Abdominal pain** | HC | 0.24±0.08 | 0.14±0.06 | 0.13±0.05 | 0.18±0.07 | main effect of group p<0.05  (FD>HC) |
|  | FD | 1.93±0.75* | 0.82±0.39 | 0.90±0.31* | 1.54±0.60* |  |
| **Abdominal discomfort** | HC | 0.21±0.06 | 0.14±0.06 | 0.22±0.10 | 0.21±0.10 | main effect of group p<0.01  (FD>HC) |
|  | FD | 3.83±0.83*** | 3.04±0.80** | 2.53±0.75** | 2.64±0.67*** |  |
| **Burning** | HC | 0.50±0.20 | 0.21±0.08 | 0.22±0.10 | 0.18±0.07 | main effect of group p<0.01  (FD>HC) |
|  | FD | 2.47±0.97*** | 1.39±0.62** | 1.17±0.64** | 2.93±0.82*** |  |
| **Bloating** | HC | 0.29±0.13 | 0.39±0.16 | 0.38±0.17 | 0.41±0.19 | main effect of time p<0.05  main effect of group p<0.01  (FD>HC) |
|  | FD | 2.50±0.83* | 4.43±0.87*** | 3.07±0.77** | 4.32±0.83*** |  |
| **Nausea** | HC | 0.53±0.20 | 0.78±0.06 | 0.31±0.15 | 0.29±0.14 | main effect of time p<0.05 |
|  | FD | 1.47±0.66 | 1.07±0.64 | 1.23±0.41* | 1.64±0.62* |  |
| **Vomiting** | HC | 0.18±0.06 | 0.14±0.06 | 0.38±0.21 | 0.24±0.10 | Not significant |
|  | FD | 0.77±0.33 | 1.07±0.64 | 0.77±0.26 | 0.71±0.22* |  |

Mean±standard error

ANOVA: analysis of variance; Baseline: baseline VAS rating before breakfast; HC: healthy controls; Post1: VAS rating after breakfast; Post2: VAS rating 20-25 minutes after breakfast; Post3: VAS rating 45-50 minutes after breakfast; FD: functional dyspepsia patients

*, **, ***: two sample t-test FD vs HC. p>0.05, >0.01, >0.001, respectively

**Supplementary Table 2. Physiological response to and pleasantness rating of** emotional and food images in FD patients and healthy controls

|  |  | **non-food emotional images** | | | **food images** | | **P value (ANOVA)** | |
| --- | --- | --- | --- | --- | --- | --- | --- | --- |
|  |  | **Neutral** | **Positive** | **Negative** | **High -fat** | **Low**  **-fat** | **General effect (2X5)** | **Fat effect (2X2)** |
| **Pleasantness** | HC | 5.08±  0.39 | 7.46±  0.38 | 2.17±  0.18 | 6.61±0.40 | 7.38±  0.27 | Main effect of image p<0.001 | Main effect of group p<0.05  Main effect of image p<0.05 |
|  | FD | 5.27±  0.52 | 7.94±  0.30 | 1.97±  0.20 | 5.62±0.47 | 6.79±  0.39 |  |  |
| **SCR (ratio)** | HC | 0.92±  0.17 | 0.61±  0.11 | 1.69±  0.27 | 0.88±0.12 | 0.83±  0.16 | Main effect of image p<0.001 | NS |
|  | FD | 0.85±  0.17 | 0.83±  0.24 | 1.55±  0.24 | 0.72±0.08 | 1.05±  0.17 |  |  |
| **EMG_corrugator supercilii (ratio)** | HC | 1.03±  0.19 | 0.26±  0.10 | 1.96±  0.39 | 0.90±0.17 | 0.69±  0.20 | Main effect of image p<0.001 | NS |
|  | FD | 0.94±  0.21 | 0.77±  0.20 | 1.69±  0.31 | 0.66±0.18 | 0.91±  0.15 |  |  |
| **EMG_zygomaticus major (ratio)** | HC | 0.90±  0.17* | 1.35±  0.25 | 0.41±  0.10 | 1.67± 0.35 | 0.59±  0.14 | Main effect of image p<0.001  Interaction effect of group*image p<0.05 | Main effect of image p<0.01 |
|  | FD | 0.37±  0.08 | 2.00±  0.38 | 0.40±  0.11 | 0.89±  0.26 | 0.52±  0.12 |  |  |
| **HRV_SDNN (logms)** | HC | 28.79±3.81 | 25.78±  2.51 | 25.79±  2.50 | 25.90±3.01 | 25.04±  2.86 | Main effect of group p<0.05 | NS |
|  | FD | 31.52±4.53 | 32.32±  4.66 | 27.14±  4.75 | 32.06±5.02 | 35.43±  5.37 |  |  |
| **HRV_HF** | HC | 383.86±96.79 | 236.05  ±42.07 | 309.05  ±56.23 | 331.37  ±60.36 | 235.21  ±42.00 | NS | NS |
|  | FD | 401.50  ±82.67 | 338.32  ±109.64 | 261.48  ±108.06 | 298.93  ±83.37 | 459.14  ±143.18 |  |  |
| **HRV_LF/HF ratio** | HC | 1.75±  0.42 | 1.44±  0.21 | 1.38±  0.31 | 1.06±  0.13 | 1.67±  0.31 | Main effect of group p<0.05 | NS |
|  | FD | 1.02±  0.17 | 1.21±  0.25 | 0.48±  0.07* | 0.92±  0.21 | 0.86±  0.17 |  |  |

Mean±standard error

EMG: electromyography; FD: functional dyspepsia patients; HC: healthy controls; HF: high frequency; HRV: heart rate variability; LF: low frequency; NS: not significant; SCR: skin conductance response; SDNN: standard deviation of all normal RR intervals

*, **, ***: post-hoc analysis, FD vs HC. p>0.05, >0.01, >0.001, respectively

Supplementary Table 3. Anticipated FD symptom rating to food images

|  | | **Healthy controls** | **FD patients** | **P value (ANOVA)** |
| --- | --- | --- | --- | --- |
| **High fat food** | **Abdominal fullness** | 5.03±0.44 | 5.71±0.34 | Main effect of group p<0.01  (FD>HC) |
|  | **Satiation** | 4.91±0.51 | 5.33±0.50 |  |
|  | **Abdominal Pain** | 2.49±0.32 | 3.89±0.57* |  |
|  | **Burning** | 2.13±0.35 | 4.13±0.54** |  |
| **Low fat food** | **Abdominal fullness** | 5.02±0.54 | 5.39±0.46 | Not significant |
|  | **Satiation** | 4.82±0.47 | 5.07±0.58 |  |
|  | **Abdominal Pain** | 2.96±0.61 | 3.34±0.55 |  |
|  | **Burning** | 2.47±0.52 | 3.36±0.51 |  |

Mean±standard error

ANOVA: analysis of variance; FD: functional dyspepsia; HC: healthy controls

*, **, ***: post-hoc analysis, FD vs HC. p>0.05, >0.01, >0.001, respectively
